# Technical-tactical evolution of women’s football: a comparative analysis of ball possessions in the FIFA Women’s World Cup France 2019 and Australia & New Zealand 2023

**DOI:** 10.5114/biolsport.2025.139077

**Published:** 2024-05-07

**Authors:** Iyán Iván-Baragaño, Rubén Maneiro, José L. Losada, Antonio Ardá

**Affiliations:** 1 Faculty of Sports Sciences, Universidad Europea de Madrid, Madrid, Spain; 2 Faculty of Education and Sport, University of Vigo, Vigo, Spain; 3 Department of Social Psychology and Quantitative Psychology, University of Barcelona, Barcelona, Spain; 4 Department of Physical and Sport Education, University of A Coruña, A Coruña, Spain

**Keywords:** Women’s football, Key performance indicators, ball possession, FIFA Women’s World Cup, Build up, Maintenance

## Abstract

The aim of this study was to analyse and compare, both individually and multivariately, the technical-tactical similarities and differences associated with the offensive phase between the FIFA Women’s World Cup France 2019 and the FIFA Women’s World Cup Australia & New Zealand 2023. Following an observational follow-up design, 4,669 ball possessions were analysed in both tournaments (FWWC19: n = 2,323; FWWC23: n = 2,346). Differences between the two editions were examined using the chi-square statistic (p < .05) and Student’s t-test for categorical and continuous criteria, respectively. The effect size was calculated using the contingency coefficient and Cohen’s d, respectively. Lastly, a decision tree model was implemented with FWWC as the objective criterion. Statistically significant differences were found between the two tournaments for the criteria Match Status, Match Outcome, Defensive Intention, Interaction Context, MD, MO, Possession Time, and Passes. At the multivariate level, the predictor criteria introduced by the decision tree model were Match Status, Time, MO, Start Zone (width), Passes, Defensive Intention, and Possession zone. Between the two tournaments, increases in average possession time and the number of passes were observed, conditioned by greater technical and tactical efficiency of the teams. Similarly, in the latest edition, there was greater parity among the analysed teams, justifying the inclusion of more teams in the FWWC23. The results of this study demonstrate that in the last 4 years, elite women’s football has undergone a change characterized by ball possession and game control.

## INTRODUCTION

The first FIFA Women’s World Cup (FWWC) took place in China in 1991 [1], and to date, only 9 editions of this championship have been contested. This, coupled with the growth period that women’s football is experiencing in terms of the number of players and economic investment [2], has led to improvements in technical, tactical, and physical aspects over short periods of time [3, 4]. For instance, the physical report published on the penultimate edition of the FWWC France 2019 [3] noted an increase in the maximum speeds of the fastest players by approximately 2 km/h compared to the previous edition, a change that has also been observed in the edition held in Australia and New Zealand [5].

Similarly, the increase in resources from federations and clubs for women’s football has enhanced the professionalization of the sport, elevating the technical and tactical level of the players. In this regard, the European continent has seen on the field the commitment to youth women’s football, and the FWWC Australia & New Zealand 2023 has marked a turning point in the hegemony of the United States in global women’s football. In 2019, 40% of registered female players worldwide were from the United States [6], while in 2023, 72% of players under the age of 20 were registered with UEFA federations [2], clearly reflecting how the European continent leads in women’s academy football. In relation to these technical and tactical indicators, for example, the average passing accuracy in the 2011, 2015, and 2019 World Cups was 69%, 71%, and 74%, respectively [7]. In the edition of Australia and New Zealand 2023, Spain, the winning team, averaged an 86.58% passing accuracy, completing an average of 572 successful passes per game [8]. This surpassed the best passing accuracy observed four years earlier (Japan – 82%) by 4 percentage points.

**FIG. 1:**
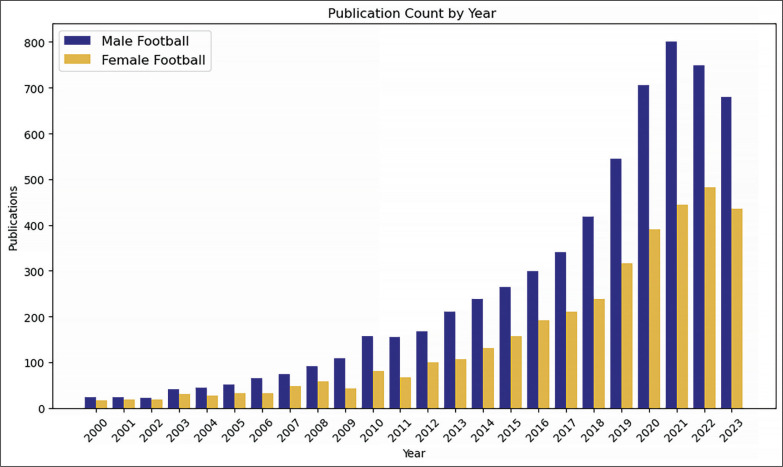
Evolution of men´s football and women´s football as research topic from 2000

Undoubtedly, the increase in research on performance indicators in women’s football, from technical, tactical, and conditioning perspectives, has also contributed to the professionalization of players – in Figure 1, the evolution in the number of publications on women’s football (“female football” OR “women’s soccer”) and men’s football (“male football” OR “men’s soccer”) from the year 2000 to the year 2023 is presented, consulted on 17/03/2024 in Web of Science. Based on that, research focused on understanding the influence of contextual variables in women’s football, such as home advantage [9], match status [10, 11], or the quality of opposition [12], has helped comprehend individual and collective behaviour based on different moments and contexts of the match. Similarly, the efforts of various researchers to expand the scientific knowledge base on aspects related to the internal and external load of players [13–16] have facilitated the adaptation of more specific training tasks and situations in women’s football.

In direct relation to the technical and tactical performance indicators in women’s football, Scanlan et al. [17] investigated the tactical criteria determining goal-scoring opportunities in the FWWC Canada 2015, similar to the studies by Iván-Baragaño et al. and Maneiro et al. [18, 19], which proposed a model of offensive success for ball possessions in women’s football, using the last World Cups played in 2015 and 2019 as a sample. On the other hand, Casal et al. [20] analysed how the participation of goalkeepers influenced the development of ball possessions in the Spanish Women’s League, and similarly, Errekagorri et al. [21] conducted a case study on a team in the second women’s division, analysing collective performance through the integration of tactical and conditioning variables. In a similar vein, a recent study [22] analysed technical and tactical differences using event data obtained from the U.S. women’s soccer team, observing statistically significant differences compared to others in the distance between the defensive line and the goal line in defensive pressure. Lastly, in direct relation to the development of ball possessions in women’s football, Dipple et al. [23] analysed the influence of variables related to passes executed in the National Women’s Super League in the United States and the Football Association Women’s Super League in England, noting that winning teams exhibited, among other characteristics, a higher number of total passes and successful passes in the final third. For these reasons, and considering that research on technical and tactical indicators in women’s football dates back only a few years and the analysed samples are still limited, this study was conducted.

Thus, with the aim of deepening our understanding of the evolution of elite women’s football in the last 4 years, the objective of this study was to analyse and compare, both individually and multivariately, the technical-tactical similarities and differences associated with the offensive phase between the FIFA Women’s World Cup France 2019 and the FIFA Women’s World Cup Australia & New Zealand 2023.

## MATERIALS AND METHODS

### Design

To carry out this study, an observational methodology [24] was employed, utilizing a nomothetic (various units of analysis corresponding to each of the teams and championships analysed), punctual (involving intrasessional follow-up throughout each of the matches), and multidimensional (various levels of response reflected in the observation instrument) design. This design corresponds to the third observational quadrant proposed by Anguera et al. [25]. All analysed matches were recorded from public television, stored on an external hard drive, and analysed after the event. According to the Belmont Report [26], the use of publicly available images for research purposes does not require informed consent from participants or approval from an ethics committee

### Participants

A total of 4,669 ball possessions were analysed (n-FWWC19 = 2.323; n-FWWC23 = 2.346) in the 32 matches (16 matches per championship) corresponding to the knockout phase of the FIFA Women’s World Cup 2019 and 2023. Each of the teams was considered as a unit of analysis, and thus their technical-tactical behaviour was analysed as a unit. The inclusion criteria for the analysis of possessions consisted of: i) two consecutive contacts by the same player with the ball, or ii) a completed pass, or iii) a shot taken, provided that the duration was equal to or greater than 4 s [17]. To homogenize the analysed sample, ball possessions that took place during extra time were excluded.

### Observation instrument

The observation instrument utilized for this study was proposed by Iván-Baragaño et al. [17] and can be referred to in Table 1. The instrument was developed *ad hoc* by a committee of experts in football and consisted of a combination of field format and category systems. In total, 17 criteria related to the start, development, and outcome of ball possessions were analysed. Among the criteria related to the start of possessions, contextual criteria such as match outcome, match status or temporality were introduced, as well as spatial criteria related to the start zone of possessions. The development of possessions was based on the analysis of criteria such as offensive and defensive tactical intention or the duration of possessions, as well as the number of passes. Finally, the outcome of possessions was recorded as Goal, Shot, Pass into Area, or Unsuccessful.

**TABLE 1:** Observation Instrument

Criteria	Categories	Description
FWWC	FWWC19	FIFA Women´s World Cup France 2019
	FWWC23	FIFA Women´s World Cup Australia & New Zealand 2023

Match Outcome	Win	The team observed won the match
	Lose	The team observed lost the match
	Draw	The team observed draw the match

Time	1Q	Possession starts between the start of the game and minute 15
	2Q	Possession starts between minute 16 and minute 30
	3Q	Possession starts between minute 31 and the end of the first half
	4Q	Possession starts between the start of the second half and minute 60
	5Q	Possession starts between minute 61 and minute 75
	6Q	Possession starts between minute 76 and the end of the game

Match Status	Winning	The team observed is winning when the action starts
	Drawing	The teams are level when the action starts
	Losing	The team observed is losing when the action starts

Start Form	Set Play	Possession begins after a regulatory interruption of the game.
	Transition	Possession begins without a regulatory interruption.

Start Zone (length)	Defensive	Possession begins in the defensive area of the pitch
	Predefensive	Possession begins in the predefensive area of the pitch
	Middle	Possession begins in the middle area of the pitch
	Preoffensive	Possession begins in the preoffensive area of the pitch
	Offensive	Possession begins in the offensive area of the pitch

Start Zone (width)	Left	Possession starts from the left wing
	Central	Possession starts from the center
	Right	Possession starts from the right wing

Defensive Organization	Organised	The opposing team is defensively organised
	Circumstantial	The opposing team is defensively disorganised

Defensive Positioning	Low	Opponents positioning is at the back at the start of the action
	Medium	Opponents positioning is midfield at the start of the action
	Advanced	Opponents positioning is forward at the start of the action

Interaction Context	MM	Midfield zone vs midfield zone
	RA	Rear zone vs forward zone
	RM	Rear zone vs midfield zone
	A0	Forward zone vs goalkeeper
	AA	Forward zone vs forward zone
	AM	Forward zone vs midfield
	AR	Forward zone vs rear zone
	MA	Midfield zone vs forward zone
	MR	Midfield zone vs rear zone
	PA	Goalkeeper vs forward zone

Offensive Intention	Keep	The team observed tries to maintain possession of the ball
	Progress	The team observed tries to progress towards the rival goal

Defensive Intention	No pressure	The opposing team shows an intention to defend their goal
	Pressure	The opposing team shows an intention to recover the ball

MD (seconds)		Time of possession in own half (in seconds)

MO (seconds)		Time of possession in opponent´s half (in seconds)

Possession Time		Total time of possession

Passes		Number of passes

Possession Zone	MD	Most possession in own half
	MO	Most possession in opponent´s half

Possession Outcome	Goal	The possession ends with a goal
	Shot	The possession ends with a shot
	Sent to Area	The possession ends with a ball into the penalty area
	No Succes	The possession ends with no success.

**FIG. 2:**
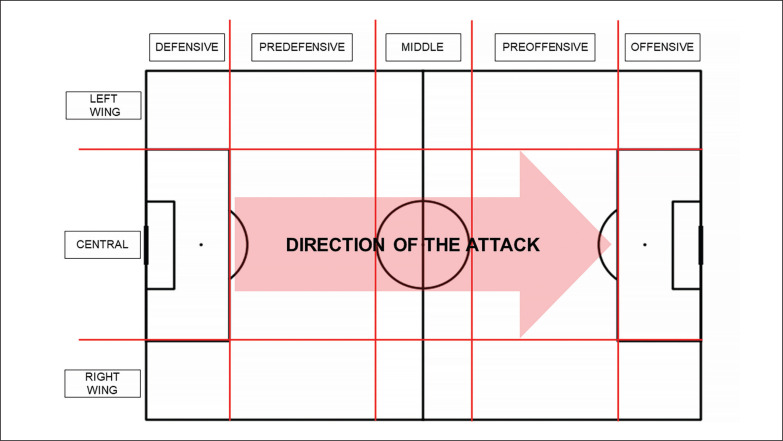
Game space zoning used in this study.

The recording tool used was the open-source software LINCE PLUS V 1.3.2. [27]. The criteria “start zone (length)” and “start zone (width)” were recorded as presented in Figure 2.

### Procedure and reliability

Prior to the recording and coding of all actions, three observers were trained and familiarized with the observation instrument following the procedure proposed by Losada & Manolov [28]. Two of them held doctoral degrees in Sports Science with over 30 years of combined experience in observational methodology, and the third was a Ph.D. student. All three possessed the UEFA PRO coaching license. Data quality control was conducted using Cohen’s kappa coefficient [29], obtaining an average value from the pairs of 0.869, considered excellent according to the scale of Landis & Koch [30]. This average was calculated as both the inter-observer and intra-observer value after recording a total of 258 ball possessions from two randomly selected matches. The researcher responsible for recording the possessions analysed the initial sample (n = 258) twice to verify consistency in the recording. The results of the data quality control for each of the analysed criteria are presented in Table 2.

**TABLE 2:** Reliability values obtained for the criteria include in the observation instrument

Criteria	Cohen’s Kappa Average
Match Outcome	.886
Time	.979
Match Status	.936
Start Form	.890
Start Zone (length)	.897
Start Zone (width)	.904
Defensive Organization	.746
Defensive Positioning	.853
Interaction Context	.817
Offensive Intention	.846
Defensive Intention	.726
MD (seconds)	.957
MO (seconds)	.916
Possession Time	.969
Passes	.966
Possession Zone	.894
Possession Outcome	.819
Kappa Overall	**.869**

### Data analysis

Firstly, a descriptive and comparative analysis was conducted through frequency counts for the two categories of the FWWC criterion (2019 & 2023). The existence of statistically significant differences was tested using the chi-square statistic, and the effect size was quantified using the contingency coefficient. The effect size was categorized as small (ES = 0.10), medium (ES = 0.30), and large (ES = 0.50) (30). For the variables MD (seconds), MO (seconds), Possession Time, and Passes, the independent samples t-test was applied, justified by the central limit theorem due to the large sample size. Prior to this, the distribution of each variable and group, as well as the presence of outliers, was assessed through graphical representation. The effect size for these four criteria was calculated as the difference between the standardized means of each, categorized in the same manner as before [31].

To address the second part of the objective, a decision tree analysis was conducted using FWWC as the dependent variable. Before selecting the final hyperparameters, various partitions and sample possibilities were preliminarily tested to avoid overfitting and under-fitting and enhance the model’s accuracy. For the final model, 70% of the observations were randomly selected as the training sample, and the remaining 30% were used as the validation sample. The other analysed criteria were introduced into the model as predictors or independents. The tree growth method used was chi-square automatic interaction detection (CHAID). The statistical significance value for creating new nodes was set at p < .05, with a minimum of 100 observations for parent nodes and 50 for end nodes. The maximum depth of the decision tree was set at 4 levels. Lastly, the model’s validity was evaluated using the correct classification table (false positives/false negatives) and the area under the ROC curve (AUC), considered excellent (0.90 < AUC < 1.00), good (0.80 < AUC < 0.90), fair (0.70 < AUC < 0.8), poor (0.6 < AUC < 0.7), and fail (0.5 < AUC < 0.6) [32].

All analyses were conducted using SPSS 26.0 statistical software (IBM Corp., Released 2017. IBM SPSS Statistics for Windows, Version 26, IBM Corp., Armonk, NY, USA).

## RESULTS

**FIG. 3:**
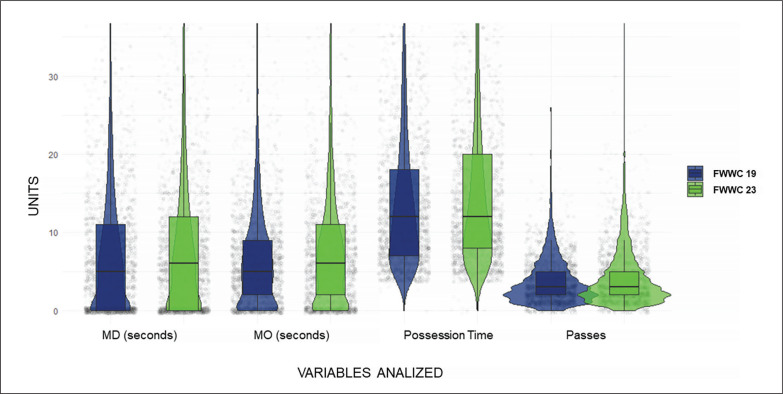
Graphical representation of the variables MD (seconds), MO (seconds), Possession Time, and Passes.

The descriptive and bivariate results are presented in Table 3. Statistically significant differences were found between the two analysed championships in the criteria Match Outcome (*p* < .001; ES = .09), Match Status (*p* < .001; ES = .135), Interaction Context (*p* < .001; ES = .09), and Defensive Intention (*p* < .001; ES = .07). Additionally, a significant increase was observed in the distribution of the 4 analysed quantitative variables: MD (seconds) (*p* < .001; ES = .11), MO (seconds) (*p* < .001; ES = .13); Possession Time (*p* < .001; ES = .18), and Passes (*p* < .001; ES = .16), as depicted in Figure 3.

**TABLE 3:** Descriptive and bivariate results

		FWWC2019 n = 2323	FWWC2023 n = 2346	p overall
Match Outcome	Win	903 (38.9%)	935 (39.9%)	**< .001 [.09]**
	**Draw**	440 (18.9%)^**^	594 (25.3%)^*^	
	**Lose**	980 (42.2%)^*^	817 (34.8%)^**^	

Time	1Q	410 (17.6%)	415 (17.7%)	= .862
	2Q	393 (16.9%)	365 (15.6%)	
	3Q	403 (17.3%)	418 (17.8%)	
	4Q	367 (15.8%)	385 (16.4%)	
	5Q	365 (15.7%)	364 (15.5%)	
	6Q	385 (16.6%)	399 (17.0%)	

Match Status	Winning	540 (23.2%)	522 (22.3%)	**< .001 [.135]**
	Drawing	978 (42.1%)^**^	1273 (54.3%)^*^	
	Losing	805 (34.7%)^*^	551 (22.5%)^**^	

Start Form	Set Play	734 (31.6%)	759 (32.4%)	= .521
	Transition	1588 (68.4%)	1587 (67.6%)	

Start Zone (length)	Defensive	366 (15.8%)	387 (16.5%)	= .213
	Predefensive	771 (33.2%)	726 (30.9%)	
	Middle	625 (26.9%)	616 (26.3%)	
	Preoffensive	485 (20.9%)	519 (22.1%)	
	Offensive	76 (3.3%)	98 (4.2%)	

Start Zone (width)	Left	526 (22.6%)	588 (25.1%)	= .113
	Central	1234 (53.1%)	1186 (50.6%)	
	Right	563 (24.2%)	572 (24.4%)	

Defensive	Organized	2256 (97.1%)	2310 (98.5%)	**< .005 [.05]**
Organization	Circumstantial	63 (2.7%)	36 (1.5%)	

Defensive Positioning	Advanced	854 (36.8%)	879 (37.5%)	= .156
	Medium	416 (17.9%)	451 (19.2%)	
	Low	1050 (45.2%)	1016 (43.3%)	

Interaction Context	MM	943 (40.6%)	892 (38.0%)	**< .001 [.09]**
	RA	739 (31.8%)^**^	865 (36.9%)^*^	
	RM	78 (3.4%)^*^	55 (2.3%)^**^	
	A0	10 (0.4%)^*^	1 (0.04%)^**^	
	AA	39 (1.7%)	35 (1.5%)	
	AM	15 (0.6%)	22 (0.9%)	
	AR	193 (8.3%)	178 (7.6%)	
	MA	29 (1.2%)	46 (2.0%)	
	MR	37 (1.6%)	16 (0.7%)	
	PA	238 (10.2%)	236 (10.1%)	

Offensive Intention	Keep	1347 (58.0%)	1343 (57.2%)	= .609
	Progress	976 (42.0%)	1003 (42.8%)	

Defensive Intention	No Pressure	1448 (62.3%)^*^	1325 (56.5%)^**^	**< .001 [.07]**
	Pressure	872 (37.5%)^**^	1021 (43.5%)^*^	

MD (seconds)		7.24 ± 7.76	8.15 ± 9.24	**< .001 [0.11]**

MO (seconds)		6.75 ± 6.51	7.75 ± 8.22	**< .001 [0.13]**

Possession Time		13.93 ± 8.99	15.82 ± 12.13	**< .001 [0.18]**

Passes		3.64 ± 2.85	4.19 ± 3.8	**< .001 [0.16]**

Possession Outcome	Goal	26 (1.1%)	30 (1.3%)	= .101
	Shot	209 (9.0%)	166 (7.1%)	
	Sent to Area	344 (14.8%)	343 (14.6%)	
	No Success	1744 (75.1%)	1807 (77.9%)	

Note.

* More observed than expected values obtained through the normalized adjusted residual,

** Less observed than expected values through the normalized adjusted residual.

**FIG. 4:**
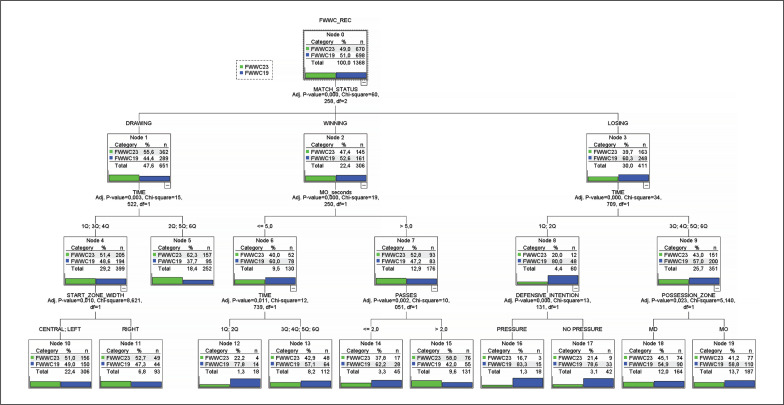
Classification model based on the decision tree algorithm

Regarding the decision tree model (Figure 4), the criteria introduced by the algorithm were: i) Match Status, ii) Time, iii) MO (seconds), iv) Start Zone (width), v) Passes, vi) Defensive Intention, and vii) Possession zone. The probabilities assigned to each category of the FWWC criterion (2019 vs 2023) can be consulted in Figure 3. Among the most notable results were the following. Node 0 consisted of a total of 1,368 observations (30% of the sample), with 49% corresponding to FWWC23 and 51% to FWWC19. The first predictor introduced by the algorithm was Match Status (*χ*^2^ = 60.258; df = 2, p < .001), resulting in the three main branches of the decision tree for the categories Drawing (Node 1: FWWC23 = 55.6%; FWWC19 = 44.4%), Winning (Node 2: FWWC23 = 47.4%; FWWC19 = 52.6%), and Losing (Node 3: FWWC23 = 39.7%; FWWC19 = 60.3%). Continuing the reading of the tree along the central branch, from Node 2 (Match Status = Winning), the next criterion introduced by the decision tree was MO (seconds) (*χ*^2^ = 10.250; df = 1, p < .001). Thus, Node 6 (Match Status = Winning & MO (seconds) ≤ 5) yielded a probability in favour of the FWWC19 category of 60% (n = 78), while Node 7, with a value greater than 5 for the MO (seconds) criterion, decreased the probability in favour of FWWC19 to 47.2%. To conclude this central branch, the next criterion introduced was Passes (*χ*^2^ = 60.258; df = 1, p < .001), the highest probability being observed in favour of the FWWC23 category for the interaction of categories: i) Match Status = Winning, MO (seconds) ≥ 5, and Passes ≥ 3 (Node 15; FWWC23 = 58.0%, FWWC19 = 42.0%). Based on the branches derived from Node 3 (Match Status = Losing), it was observed that the probability of a possession made under that match status and with a temporality of 1Q or 2Q (i.e., from the start of the match until minute 30) corresponding to the FWWC19 category was 80%, while the observed probability of a possession losing, from minute 30 onwards was: Node 9; FWWC23 = 43%, FWWC19 = 57%. The training decision tree showed a correct classification percentage of 58.1% (64.9% sensitivity (FWWC23) – 51.1% specificity (FWWC19). Finally, the validation model presented a correct classification percentage of 57.9% (65.4% sensitivity (FWWC23) – 50.7% specificity (FWWC19) and an area under the curve (AUC) equal to 0.581 (95% CI = 0.565–598), showing that despite the differences found, the classification of ball possessions based on the FWWC criterion using a decision tree model did not achieve acceptable results.

## DISCUSSION

The objective of this study was to analyse and compare, individually and multivariately, the technical-tactical similarities and differences associated with the offensive phase between the FIFA Women’s World Cup France 2019 and the FIFA Women’s World Cup Australia & New Zealand 2023. For this purpose, 4,669 ball possessions (n2019 = 2,323; n2023 = 2,346) were analysed between the two championships using observational methodology.

The average number of possessions per team and per game was 72.59 and 73.31 in the FWWC France 2019 and FWWC 2023, respectively. This result might indicate a priori that the number of losses, steals, and/or ball transitions remained stable during both championships. However, the average number of possessions in both championships clearly contradicts the results obtained in this study for the Possession Time criterion. Compared to the 2019 France edition, ball possessions were on average 13.5% longer (Possession Time FWWC19 = 13.93 s; FWWC23 = 15.82 s), which can be explained by a longer total duration and effective playing time of the matches played. Although these data have not been provided in the official reports of each match [8], the fact that matches such as Spain – Netherlands in the Quarterfinals had an extra time of 20 minutes (+30 minutes of subsequent overtime) seems to indicate that this difference reflects a trend present throughout the championship [3].

Similarly, the 13.5% increase in the average duration of ball possessions aligns with the 12.5% increase observed in this study in the time of possession in own half (from 7.24 s to 8.15 s on average between the two editions), the 14% increase in time in the opponent’s half (FWWC2019 = 6.75 s vs FWWC23 = 7.75 s), and the 15% increase in the average number of passes per analysed action (3.62 passes/possession vs 4.19 passes/possession). While statistically, there was a small or moderate effect size, from a football perspective, these results are highly important, highlighting an improvement in the quality of ball possessions based on increased technical and tactical efficiency of the participating teams [8]. Moreover, the results obtained from the decision tree analysis seem to confirm this tendency in the FIFA Women’s World Cup Australia & New Zealand 2023. In nodes 7 and 15 of the decision tree, it was observed that possessions made while winning, with a duration longer than 5 s in the opponent’s half (node 7), and more than two passes (node 15) were significantly more likely to occur in the 2023 edition compared to their sibling nodes (node 6 and node 14, respectively).

Regarding the Interaction Context criterion, statistically significant differences were also observed between the two analysed championships. While the decision tree algorithm did not introduce this criterion into the model, possibly due to the high number of categories and the consequent reduction in the number of observations per category, the differences observed in the initial interaction context could imply that teams are modifying their offensive and defensive strategies, although this will need to be studied in future research. In both FIFAWWC19 and FWWC23, the categories with the highest frequency were middle vs middle (MM) (FWWC19 = 40.6%; FWWC23 = 38.0%) and rear vs forward (RA) (FWWC19 = 31.8%; FWWC23 = 36.9%). This aligns with the decrease in the frequency of highly offensive interaction contexts such as A0 (forward vs goalkeeper) observed by Barreira et al. [33] and with the frequencies observed by Maneiro et al. [34] in the Men’s European Championships of 2008 and 2016. In this study [34], it was observed that the category with the highest frequency in men’s football in both samples was RA. Thus, a higher frequency of the MM category compared to RA (characteristic of women’s football and more frequent in FWWC19) [10, 17] might indicate greater difficulty for teams in the offensive phase and overcoming areas of higher player density. Finally, a significant reduction in the appearance of the A0 category was observed in the 2023 edition. This interaction context, often caused by a technical error by the player, was 10 times less frequent, possibly indicating an improvement in the technical (and tactical) skills of the defensive line and goalkeepers. In line with this, Kirkendall [35] stated, after interviewing women’s football coaches, that the defensive line typically showed lower technical performance in the attacking phase, and although these data could only be verified through individual performance analysis, the findings of this study also suggest that this situation is changing.

Lastly, but not less important, statistically significant differences were observed in the two analysed criteria associated with the match outcome (match status and match outcome). In the 2023 edition held in Australia and New Zealand, there was a greater prevalence of the categories Draw and Drawing, along with a decrease in the frequency of the categories Lose and Losing, corresponding to the match outcome and match status criteria, respectively. This change may be significant for various reasons. First, previous studies in both men’s [36] and women’s [10] football have demonstrated that the match status is a criterion that can modify offensive and defensive strategies. Additionally, the 2023 edition was the first to feature 32 national teams, as part of FIFA’s commitment to increasing the number of teams. Thus, the increase in teams did not lead to a greater imbalance in the analysed matches, nor does it seem to have done so in the group stage based on the number of goals scored (2.56 goals/match), which is the lowest in the history of the Women’s World Cup. Based on this criterion (Match Status), in nodes 8 and 9 of the decision tree, a clear difference between the two championships can be observed: 80% of possessions made while losing between the 0^th^ and 30^th^ minute of the match were recorded in FWWC2019, represented by this criterion interaction, among other events, in the fact that the United States managed to take the lead before the 14^th^ minute in all matches played (except the final against the Netherlands) [7].

To conclude this discussion section, it should be noted that the decision tree model, designed to assess the classification ability (and thus differentiation) between the two analysed championships, was not able to correctly classify both categories (FWWC19 and FWWC23) adequately (AUC = .581). Firstly, we must acknowledge that, while we consider the observed differences in this study between the two championships significant from a technical and tactical perspective, there would need to be more evident statistical patterns to enable accurate classification using a decision tree model. However, on the other hand, we must contextualize the reality that has been analysed. Thus, considering that the history of the FIFA Women’s World Cup began in 1991 with 12 participating teams and that progress has been continuous over the editions held, the differences found could be considered a significant change in the performance of participating teams. This change should be confirmed in future elite women’s football championships.

## CONCLUSIONS

In this study, a descriptive and comparative analysis of ball possessions in the FIFA Women’s World Cup France 2019 and the FIFA Women’s World Cup France 2023 was conducted. A total of 4,669 ball possessions were analysed between the two championships. While the number of possessions per team and per match was similar between the two championships, a statistically significant increase was observed in FWWC23 in the duration of possessions, the number of passes per possession, as well as possession time in the own and opponent’s field. These differences may be associated with a better technical and tactical performance of the teams.

On the other hand, significant differences were observed in the criteria of Interaction Context and Defensive Intention. For the former, a slight difference related to criteria with high offensive value was observed. Regarding defensive intention, there was an increase in the category Pressure, possibly related to a greater tendency shown in FWWC23 to control the game through ball possession. Finally, the differences found between the two championships for the criteria Match Status and Match Outcome could be explained by greater parity in the matches. This aligns with the fact that the number of goals per match was the lowest in the history of the World Cup, helping to explain the increase from 24 to 32 teams proposed by FIFA between the two championships.

## Conflicts of interest

The authors declare no conflicts of interest.
